# SWGTS—a platform for stream-based host DNA depletion

**DOI:** 10.1093/bioinformatics/btae332

**Published:** 2024-05-24

**Authors:** Philipp Spohr, Max Ried, Laura Kühle, Alexander Dilthey

**Affiliations:** Algorithmic Bioinformatics, Heinrich Heine University Düsseldorf, Düsseldorf, 40225, Germany; Center for Digital Medicine, Düsseldorf, 40225, Germany; Algorithmic Bioinformatics, Heinrich Heine University Düsseldorf, Düsseldorf, 40225, Germany; Center for Digital Medicine, Düsseldorf, 40225, Germany; Algorithmic Bioinformatics, Heinrich Heine University Düsseldorf, Düsseldorf, 40225, Germany; Center for Digital Medicine, Düsseldorf, 40225, Germany; Center for Digital Medicine, Düsseldorf, 40225, Germany; Institute of Medical Microbiology and Hospital Hygiene, University Hospital Düsseldorf, Heinrich Heine University Düsseldorf, Düsseldorf, 40225, Germany

## Abstract

**Motivation:**

Microbial sequencing data from clinical samples is often contaminated with human sequences, which have to be removed prior to sharing. Existing methods for human read removal, however, are applicable only after the target dataset has been retrieved in its entirety, putting the recipient at least temporarily in control of a potentially identifiable genetic dataset with potential implications under regulatory frameworks such as the GDPR. In some instances, the ability to carry out stream-based host depletion as part of the data transfer process may be preferable.

**Results:**

We present SWGTS, a client–server application for the transfer and stream-based host depletion of sequencing reads. SWGTS enforces a robust upper bound on the maximum amount of human genetic data from any one client held in memory at any point in time by storing all incoming sequencing data in a limited-size, client-specific intermediate processing buffer, and by throttling the rate of incoming data if it exceeds the speed of host depletion carried out on the SWGTS server in the background. SWGTS exposes a HTTP–REST interface, is implemented using docker-compose, Redis and traefik, and requires less than 8 Gb of RAM for deployment. We demonstrate high filtering accuracy of SWGTS; incoming data transfer rates of up to 1.65 megabases per second in a conservative configuration; and mitigation of re-identification risks by the ability to limit the number of SNPs present on a popular population-scale genotyping array covered by reads in the SWGTS buffer to a low user-defined number, such as 10 or 100.

**Availability and implementation:**

SWGTS is available on GitHub: https://github.com/AlBi-HHU/swgts (https://doi.org/10.5281/zenodo.10891052). The repository also contains a jupyter notebook that can be used to reproduce all the benchmarks used in this article. All datasets used for benchmarking are publicly available.

## 1 Introduction

The sharing of raw sequencing reads within the scientific community, e.g. publicly *via* SRA or ENA or across institutions as part of a multi-center study, is an important best practice in microbial genomics and microbiome research. Sharing of raw sequencing reads typically involves the identification and removal of “contaminant” human reads (“host depletion”); these are produced as a by-product of many sequencing workflows (Bush *et al.* 2020) and carry the risk of individual re-identification (Tomofuji *et al.* 2023). Of note, even small amounts of human genetic data are sufficient to raise privacy and identifiability concerns, as <100 SNPs may be sufficient for subject re-identification (Lin *et al.* 2004, Pakstis *et al.* 2010). Multiple effective host depletion methods have been developed, including Hostile (Constantinides *et al.* 2023), dehumanizer (https://github.com/SamStudio8/dehumanizer) or ReadItAndKeep (Hunt *et al.* 2022); in addition, general mapping or classification tools like minimap2 (Li 2018) or Kraken 2 (Wood *et al.* 2019) can also be used. Existing methods, however, only support the “bulk depletion” use case, i.e. removal of human data from a locally stored sequencing data file.

Here, we present the Secure Whole-Genome Transfer System (SWGTS), the first approach for the depletion of host reads in an incoming stream of sequencing data; by relying on a defined-size buffer for the temporary storage of incoming and as-of-yet uncontaminated sequencing data. SWGTS enforces a robust upper bound on the amount of human read data stored at any point during the transfer process. To motivate the use case for SWGTS, consider the case of a sequencing data exchange between Bob and Alice, with data flowing from Bob to Alice. Alice wants to ensure that the data received from Bob does not contain an identifiable amount of human genetic sequencing data at any point in time. For example, Alice may operate a public sequencing data archive; provide a cloud-based bioinformatics data analysis service; function as the centralized sequencing data collection node for a multi-center pathogen genome sequencing study; or want to avoid re-identifiability and legal risks associated with human genetic data potentially arising under the GDPR (Shabani and Marelli 2019). Alice could ask Bob to carry out human decontamination prior to transmitting any data, but whether and how Bob actually implements decontamination is out of Alice’s control. Alice may therefore implement her own decontamination process, but existing “bulk” approaches only become applicable after receipt of the complete dataset. If Bob’s data are sufficiently contaminated, Alice will thus—at least for the period of time until her own decontamination process is successfully executed, which may vary depending on available system resources or be affected by unforeseen technical failures—be in control of identifiable human genetic data. Using SWGTS, however, Alice can set the size of the buffer for incoming sequencing data to appropriately minimize re-identification risks even if the un-decontaminated data held in memory was exclusively human; using SWGTS, Alice thus ensures that she is never in control of a personally identifiable human genetic sequencing dataset.

SWGTS implements a minimap2-based approach for the identification of human reads and carries out depletion by removal of sequences that align against the human genome (“Host Subtraction”); by exclusive retention of sequences that align against a target pathogen genome (“Simple Pathogen Retention”); or by exclusive retention of sequences that align to a target pathogen genome where the reference being aligned to also includes the human genome (“Host-Competitive Pathogen Retention”).

## 2 Materials and methods

### 2.1 Implementation

SWGTS is based on a client–server architecture, implemented using docker-compose. The SWGTS client splits a locally stored sequencing read dataset into equally-sized packets of a defined size (“chunks”) and sequentially transmits these to the SWGTS server. For each ongoing upload, the SWGTS server maintains an in-memory “buffer” of a configured maximum size of reads that have not yet undergone host depletion; an incoming chunk of reads is accepted if and only if the contained reads can be added to the buffer without the buffer exceeding its specified maximum size, and otherwise the chunk is rejected. Host depletion is implemented as a continuously running background process on the server, based on mapping the chunks sent by the client with the in-memory “mappy” version of minimap2 against the human reference genome (“Host Subtraction”); the target pathogen genome (“Simple Pathogen Retention”); or against the human reference genome plus the target pathogen genome (“Host-Competitive Pathogen Retention”). Filtering criteria in different modes are summarized in Supplementary Table S2. Once a chunk has been sent to mappy, it is removed from the buffer; filtered reads are always deleted; non-filtered reads can be stored on the SWGTS server and the IDs of these reads can also be transmitted back to the sending client. The communication between client and server is implemented using a traefik (https://traefik.io/)-based HTTP/REST interface; redis (https://redis.io/) is used for in-memory storage on the SWGTS server. SWGTS comes with a Python-based CLI client and with a React-based browser demo client application. The most important configurable parameters include the maximum size of the buffer on the server side and the number of threads used for host depletion. A full specification of all parameters and of the REST API is given in the SWGTS repository.

### Evaluation

Four “contaminated” pathogen datasets were created representing the possible combinations of SARS-CoV-2 and multi-resistant *Staphylococcus aureus* (MRSA) as well as of Illumina (1000 000 reads per dataset) and Oxford Nanopore Technologies (ONT; 250 000 reads per dataset). For each dataset, 99% pathogen reads sampled from three different pathogen isolates (Walker *et al.* 2022) were mixed with 1% human reads sampled from three 1000 Genomes Project (1000 Genomes Project Consortium *et al.* 2015) samples; see Supplementary Note S2 for datasets and accessions. Hostile was used with its human-t2t-hla reference which was also utilized for SWGTS in “Host-Competitive Pathogen Retention” and “Host Subtraction” mode. For SARS-CoV-2 we used the MN908947.3 reference without the poly-A tail provided by ReadItAndKeep and for MRSA the assembly GCF_000013425.1 of *Staphylococcus aureus* subsp. NCTC 8325. For measuring data transfer rates, two systems were used. System A is a server system with an AMD EPYC 7742 64-Core Processor with 128 Threads and 1 TiB RAM. System B is a VM configured with 10 virtual cores using an Intel^®^ Xeon^®^ CPU E5-2618L v4 @ 2.20 GHz with 20 GB RAM and 1*GbE uplink.

### Relationship between SWGTS buffer size and re-identifiability

**Figure 1.**
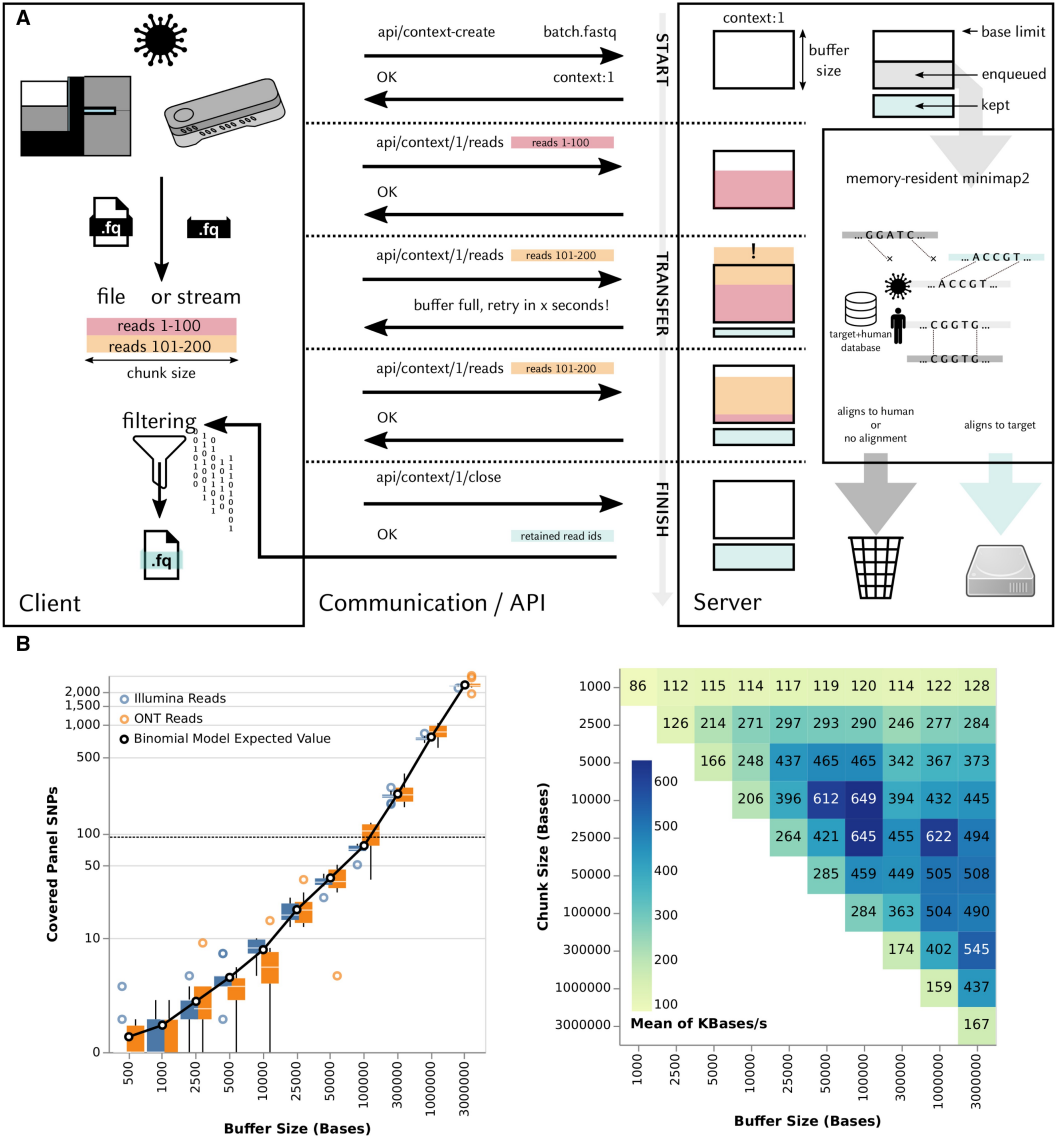
(A) Overview of SWGTS. SWGTS is a client–server application for the transfer and stream-based depletion of sequencing data from human reads. An upload to the SWGTS server is initiated by a context creation request; on the SWGTS server, each context corresponds to a unique buffer of unprocessed sequencing data of a defined maximum size (buffer size). After creation of the context, the client sequentially transmits equally-sized packets of sequencing data (chunk size) to the server, including the ID of the context just created; the server accepts chunks and adds the reads contained therein to the buffer corresponding to the specified context ID, for as long as doing so would not lead to the buffer exceeding its specified maximum size; otherwise, a “buffer full, retry in × seconds” notification is sent to the client. Single reads that are larger than the buffer are sent but immediately rejected and treated as filtered. A (multi-threaded) background process on the server continuously pulls reads from the buffer and carries out host depletion by aligning the received reads against a combined reference of the human genome and the target pathogen (in “Host-Competitive Pathogen Retention” mode). Reads that map to the pathogen are saved to disk; reads mapping to either the human reference or not at all are discarded. When a context is closed, the server sends a summary containing the IDs of the reads that were not discarded to the client. (B) Impact of the “buffer size” parameter on potential re-identifiability and performance. (Left panel) Relationship between buffer size and the number of Illumina Infinium Omni2.5-8 Kit Panel SNPs, which is pragmatically treated as a proxy for re-identifiability, covered by reads in the SWGTS buffer under worst-case assumptions (only human reads submitted); shown are empirical distributions based on real Illumina and ONT reads from three human samples (see Section 2; 10 replicates), as well as the expected value of the number of such SNPs under an approximate statistical model (“Binomial Model Expected Value”, see Supplementary Note S1). (Right panel) Relationship between buffer size and mean transfer rate for sequencing datasets between two systems, averaged over the four “contaminated” datasets representing SARS-CoV-2 and MRSA as well as Illumina and ONT (see Section 2) and in “host subtraction” mode.

The number of SNP positions present on a common population-scale SNP genotyping array (Illumina Infinium Omni2.5–8) covered by reads in the SWGTS buffer under worst-case assumptions (only human data submitted) was pragmatically treated as a proxy for re-identifiability risk (see Supplementary Note S1). To empirically characterize the relationship between the number of such SNPs and buffer size, we randomly selected reads from three 1000 Genomes Project samples (Supplementary Note S2) until the cumulative length of the selected reads was equal to the selected buffer size, trimming the last selected read if necessary. The collected reads were mapped to the human reference genome (GRCh38), counting the number of Infinium SNP positions covered by primary alignments (Supplementary Table S1, Fig. 1B, left panel). To complement experimental results and assist the determination of an appropriate buffer size without having to carry out extensive simulations, we developed statistical models for the expected number of such SNPs (Supplementary Note S1).

## 3 Results

First, we confirmed the accuracy of SWGTS with respect to discriminating between human and pathogen reads. At a buffer size large enough to process all reads and depending on the depletion mode, SWGTS retained between 96.5% and 100.0% of target pathogen reads and removed between 96.0% and 100% of human reads on the four “contaminated” datasets (Supplementary Fig. S1, Supplementary Table S1), consistent with the host depletion accuracy of minimap2 reported in the literature (Bush *et al.* 2020, Hunt *et al.* 2022) and very similar to the host retention and pathogen removal reads of Hostile (v.0.4.0, matching the “Host Subtraction” mode of SWGTS; the slight difference results from SWGTS requiring a mapping quality of at least 20 using default settings, this can be configured) and ReadItAndKeep (v.0.3.0, matching the “Regular Retention” mode of SWGTS). A decrease in buffer size resulted in a decrease of retained pathogen reads (and a slight increase in filtered human reads) since all reads exceeding the buffer size are immediately rejected; from a buffer size of ≥10 000 bases, both rates were within 3% of rates observed for the largest buffer size (Supplementary Fig. S1).

Second, we investigated how different buffer and transfer chunk sizes influence the performance of SWGTS by measuring how these variables affected transfer rates from System A to System B for the four “contaminated” datasets in “host subtraction” mode (Fig. 1B, Supplementary Fig. S2, Supplementary Table S1). Optimal performance across pathogens and sequencing data types was achieved (a) when chunk size was a fraction of buffer size, enabling parallel processing of chunks on the server side and, based on more frequently updated mapping rate estimates than for larger chunk sizes, a more accurate calibration of the “retry-after” messages sent to the client in case the buffer was full; (b) when the buffer was sufficiently large so that few retries were necessary; and (c) for chunk sizes ≥5000 bp and <50 000 bp.

Third, we characterized the relationship between the buffer size parameter and the number of Illumina Infinium Omni2.5–8 SNPs covered by reads in the SWGTS buffer under the worst-case assumption of only human data being submitted by the client; the number of such SNPs was pragmatically treated as a proxy for re-identifiability risk. Simulations (see Section 2) and statistical modeling (Supplementary Note S1) showed that the number of covered SNPs remained at ≤10 for a buffer size of 10 000 and ≤100 for a buffer size of 100 000 in almost all cases (Fig. 1B, Supplementary Table S1); furthermore, experimental results were highly consistent with the developed statistical model (Fig. 1B, Supplementary Note S1).

Finally, we evaluated which absolute data transfer speeds may be expected when using SWGTS, deploying an SWGTS server on System B (limited to 10 cores; two containers used for handling API requests; seven worker threads) and between 1 and 30 SWGTS clients on System A sending data simultaneously. Using a buffer size of 10 000 bases and transmitting the simulated SARS-CoV-2-containing Illumina read datasets, we observed data transfer rates (incoming data processed by System B) of up to approximately 0.4 megabases/s; using a buffer size of 100 000 bases and transmitting the simulated MRSA-containing Nanopore read datasets, we observed data transfer rates of up to approximately 1.8 megabases/s (see Supplementary Table S1 for full results). We note that the performance of SWGTS depends on multiple factors including network speed, the number of concurrent uploads, and the properties of the submitted data. Since the bulk of the required RAM is made up of the reference index, held in shared memory, and as each connection only adds a small fixed amount of memory, memory usage is generally consistent across buffer and chunk sizes and the number of incoming data streams and SWGTS can be deployed using relatively small amounts of RAM (<8 GB).

## 4 Discussion

We have presented SWGTS, the first approach for the transfer and stream-based human depletion of sequencing data. By combining a configurable-size buffer with a background host depletion process, SWGTS can enforce a robust upper bound on the maximum amount of human genetic data from any one client held in memory at any point in time.

Using conservative experiments, we have demonstrated that SWGTS can reliably keep the number of SNP positions present on a popular population-scale SNP genotyping array, which we have treated pragmatically as a proxy for re-identifiability risk, covered by reads in the SWGTS buffer below 100 or even 10. While the question of genetic identifiability is subtle, the risk of re-identification from so few (arbitrarily selected) SNPs has to be classified as low; in particular, to the best of our knowledge, no approaches have been presented that would allow for individual re-identification from 10 SNPs. Furthermore, we have shown consistency between the empirical results and a simple statistical model (Supplementary Note S1), which users may consult to identify a suitable application-dependent buffer size setting.

Using SWGTS can lead to significantly reduced data transfer rates; improving these, e.g. based on k-mer-based read prescreening, is therefore an important goal for future work. Additional potential improvements to SWGTS include the incorporation of a specialized short-read mapper; the improved handling of reads that exceed the buffer size, e.g. by splitting, chunk-wise processing and reconciliation; decoupling of transmission chunk size and mapping chunk size; and support for authentication and metadata handling mechanisms. These limitations notwithstanding, however, potential users of SWGTS may find that the benefits of a privacy-ensuring data transfer mechanism—such as only having to demonstrate conformity with data protection rules for demonstrably non-identifiable sequencing data—may outweigh the costs of a decreased data transfer rate.

## Supplementary Material

btae332_Supplementary_Data
