# Supplementary material for: SWGTS—a platform for stream-based host DNA depletion
Source: Bioinformatics. 2024 May 24;40(6):btae332. doi: 10.1093/bioinformatics/btae332 (PMC11167210; doi:10.1093/bioinformatics/btae332)
Supplement: btae332_Supplementary_Data [file btae332_supplementary_data.zip › Supplementary Note 1. Buffer Size Calibration.docx]

# Buffer Size Calibration: Approximating the desired in-memory base count limit

The statistical models presented in this Supplementary Note can inform intuitions about the relationship between the buffer size parameter of SWGTS and the risk of individual re-identifiability.

The question of which amount of genetic data is required to identify an individual is subtle; assume, for example, that an individual carries a very rare mutation, and that a read covering this rare mutation is present in the SWGTS buffer. On the one hand, this read could, in principle, be understood to substantially contribute to the identifiability of the affected individual; on the other hand, information on such a rare variant is unlikely to appear in the large-scale datasets that could be queried in the pursuit of re-identification, as the majority of these datasets are still based on array-based genotyping of relatively common SNPs.

The underlying assumption of the models presented below is therefore that the risk of re-identification arises primarily from the ability to determine, based on the sequencing read data in the SWGTS buffer, the genotypes of the relatively common SNPs targeted by common SNP genotyping arrays (e.g. the approximately ~2.4 million markers present on recent Illumina arrays), and that this risk increases with the number of such SNPs.

Throughout this Supplementary Note, we make the following simplifying assumptions. First, we assume that a single read covering a SNP is sufficient to determine its genotype. Second, reads are treated as independent sets of observations of individual bases; that is, two reads of 100 bases in length are treated as 200 observations of randomly determined positions in the genome. Third, the positions of SNPs are modeled as uniformly distributed across the genome. Note that the second and third simplifying assumptions are not relevant to the simulation experiments, which are based on the SNP positions of a real SNP genotyping array and in which reads are simulated as observations of contiguous stretches of the sampled genome.

## Definitions

Let $g$ be the size of the genome. Let $s < g$ be the size of the set of SNPs in the genome suitable for identifying an individual, e.g. the number of SNPs present on a popular SNP genotyping array (see above). Let $k\ll s$ be the number of unique SNPs from that set that are actually required to clearly identify an individual. Let $n$ be the buffer size in bases.

## Full statistical model

The probability of having observed less than $k$ distinct SNP positions after $n$ observations, can be expressed as: $P(less than k observed) =(\frac{1}{g})^{n}\sum_{j=0}^{k-1} \frac{s}{j}\sum_{x=j}^{min(g-s+j,n)} \frac{g-s}{x-j}S(n,x) x!$

Each sequence of $n$ bases has the same probability of $(\frac{1}{g})^{n}$. We sum over all cases where the observed distinct SNP count is less than $k$. For each allowed SNP count $j$ we look at all possible choices of $j$ SNP positions. For those choices we examine all possible set sizes $x$ for the set of positions that are either a chosen SNP position or a non-SNP position. This size is bound in two ways: Every SNP position needs to be in this set providing a lower bound. The set can naturally not exceed the amount of observations$n$ but is also bound by the genome size excluding the SNP positions that are not chosen. The $x-j$ non-SNP positions can be chosen from $g-s$ options. There are $S(n,x)$ ways of distributing the $n$ observations across the observed $x$ positions, where $S$ is the Stirling Number of the Second Kind. However, the positions are interchangeable and can thus be permuted in $x!$ ways.

The formula specified above could in principle be solved for $n$ and a given target probability to determine a suitable base count limit; however, doing so is numerically challenging, which motivates the simpler model presented in the next section.

## Binomial statistical model

Under typical assumed values for genome size $g$ and SNP set size $s$, it is reasonable to assume that the chance of hitting a SNP with more than one read base is low. We can therefore model the number of observed SNP positions using a binomial distribution. Fixing a desired maximum likelihood $\alpha=0.001$ of observing $o\geq k$ SNP positions, we want to determine the largest possible value for $n$ such that:

$\alpha\leq\sum_{j=0}^{k-1} \frac{n}{j}{\frac{s}{g}}^{j}(1-\frac{s}{g})^{n-j}=P(o<k)$

This value can be determined using a binary search. We note that this model is extremely conservative: Actual reads need non-SNP context in order to be mapped and useful, thus any read of fixed length in which SNPs are observed also implicitly indicates a given amount of non-SNP positions.
