# Supplementary material for: SWGTS—a platform for stream-based host DNA depletion
Source: Bioinformatics. 2024 May 24;40(6):btae332. doi: 10.1093/bioinformatics/btae332 (PMC11167210; doi:10.1093/bioinformatics/btae332)
Supplement: btae332_Supplementary_Data [file btae332_supplementary_data.zip › Supplementary Note 2. Read Data Accessions.docx]

Human Reads

NA18989

ONT:

ftp://ftp.1000genomes.ebi.ac.uk/vol1/ftp/data_collections/HGSVC3/working/20211013_ONT_Rebasecalled/NA18989/20210519_210512_21-lee-006_PCT0053_2-A9-D9_guppy-5.0.11-sup-prom_fastq_pass.fastq.gz

Illumina:

ftp://ftp.1000genomes.ebi.ac.uk/vol1/ftp/phase3/data/NA18989/sequence_read/SRR015521_1.filt.fastq.gz

ftp://ftp.1000genomes.ebi.ac.uk/vol1/ftp/phase3/data/NA18989/sequence_read/SRR015521_2.filt.fastq.gz

HG02282

ONT:

ftp://ftp.1000genomes.ebi.ac.uk/vol1/ftp/data_collections/HGSVC3/working/20211013_ONT_Rebasecalled/HG02282/20211102_211027_21-lee-006_PCT0053_2-A5-D5_guppy-5.0.11-sup-prom_fastq_pass.fastq.gz

Illumina:

ftp://ftp.1000genomes.ebi.ac.uk/vol1/ftp/phase3/data/HG02282/sequence_read/ERR055344_1.filt.fastq.gz

ftp://ftp.1000genomes.ebi.ac.uk/vol1/ftp/phase3/data/HG02282/sequence_read/ERR055344_2.filt.fastq.gz

HG00268

ONT:

ftp://ftp.1000genomes.ebi.ac.uk/vol1/ftp/data_collections/HGSVC3/working/20211013_ONT_Rebasecalled/HG00268/20210913_210831_21-lee-006_PCT0053_2-A1-D1_guppy-5.0.11-sup-prom_fastq_pass.fastq.gz

Illumina:

ftp://ftp.1000genomes.ebi.ac.uk/vol1/ftp/phase3/data/HG00268/sequence_read/ERR016221_1.filt.fastq.gz

ftp://ftp.1000genomes.ebi.ac.uk/vol1/ftp/phase3/data/HG00268/sequence_read/ERR016221_2.filt.fastq.gz

MRSA

https://www.ncbi.nlm.nih.gov/bioproject/PRJNA1012291

Isolate 1 Illumina:

SRR25890263

Isolate 1 Nanopore:

SRR25890280

Isolate 2 Illumina:

SRR25890262

Isolate 2 Nanopore:

SRR25890279

Isolate 3 Illumina:

SRR25890261

Isolate 3 Nanopore:

SRR25890268

SARS-COV2

https://www.ncbi.nlm.nih.gov/bioproject/PRJNA774115

Isolate 1 Illumina:

SRR16555926

Isolate 1 Nanopore:

SRR16555792

Isolate 2 Illumina:

SRR16555757

Isolate 2 Nanopore:

SRR16555741

Isolate 3 Illumina:

SRR16555506

Isolate 3 Nanopore:

SRR16555592
