# Supplementary figures and images for: SWGTS—a platform for stream-based host DNA depletion
Source: Bioinformatics. 2024 May 24;40(6):btae332. doi: 10.1093/bioinformatics/btae332 (PMC11167210; doi:10.1093/bioinformatics/btae332)

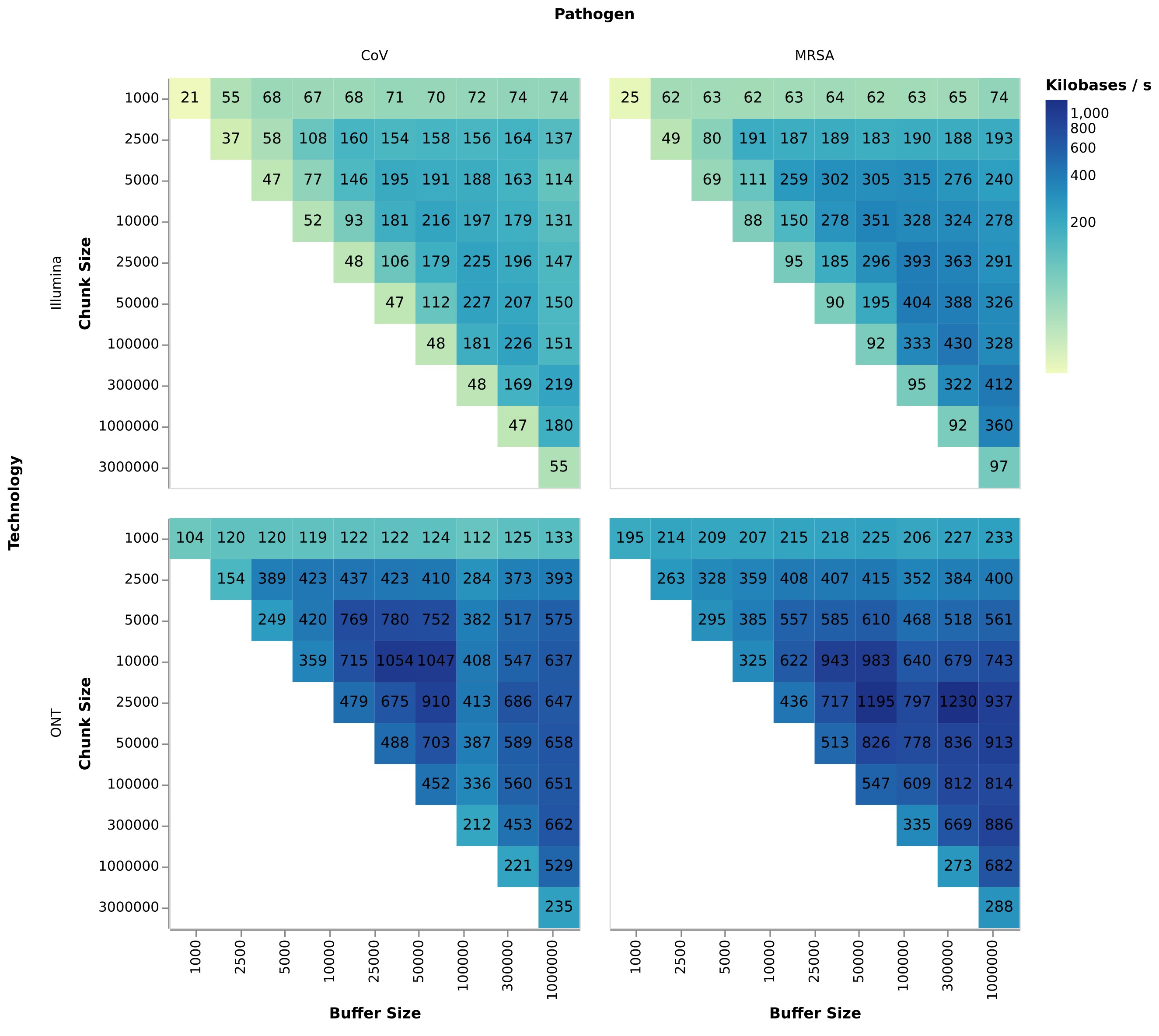

Supplement: btae332_Supplementary_Data [file btae332_supplementary_data.zip › Supplementary Figure 2. Impact of Chunk and Buffer Size.png]

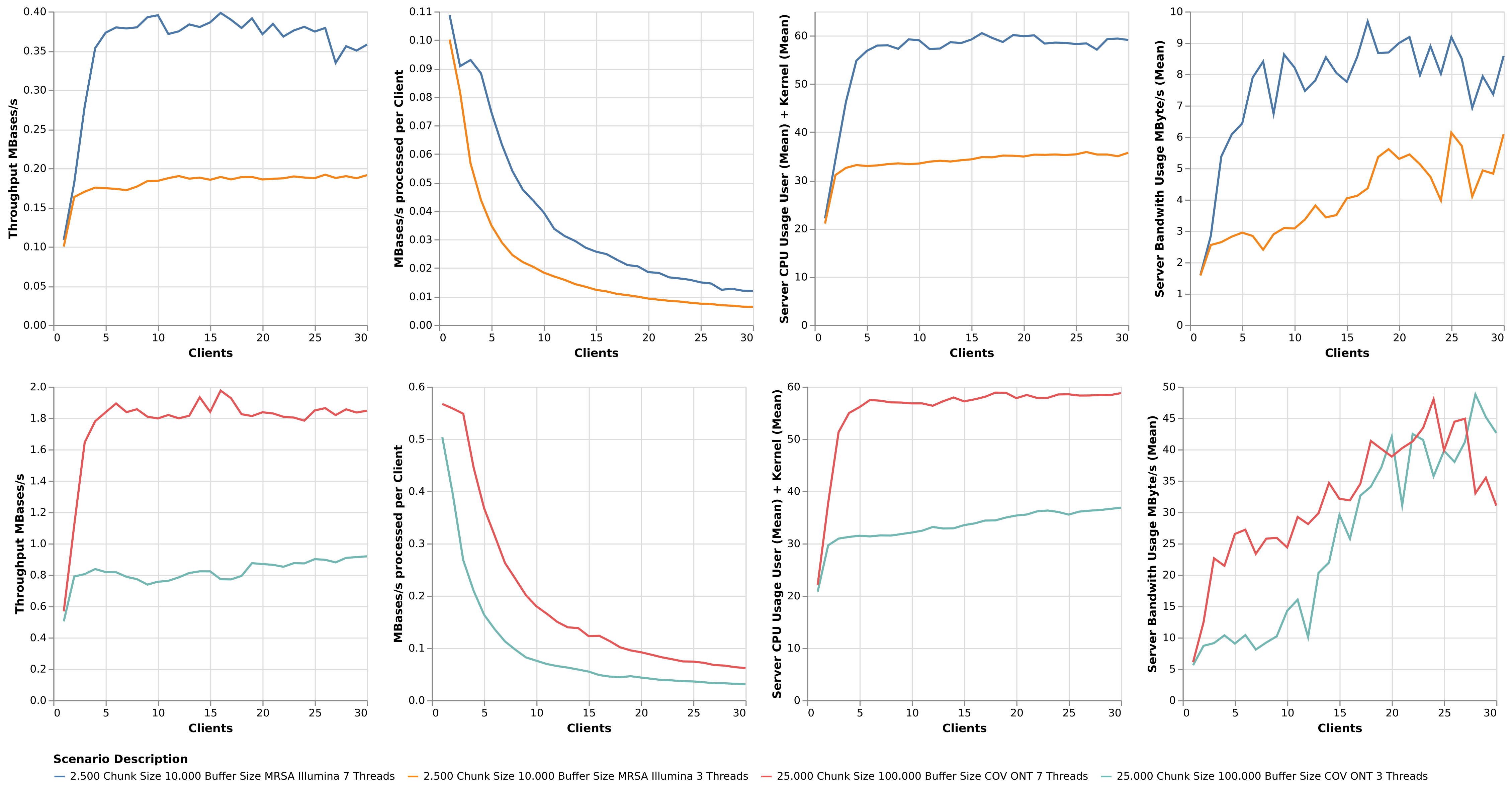

Supplement: btae332_Supplementary_Data [file btae332_supplementary_data.zip › Supplementary Figure 3. Transmission Rates and Client Scaling.png]

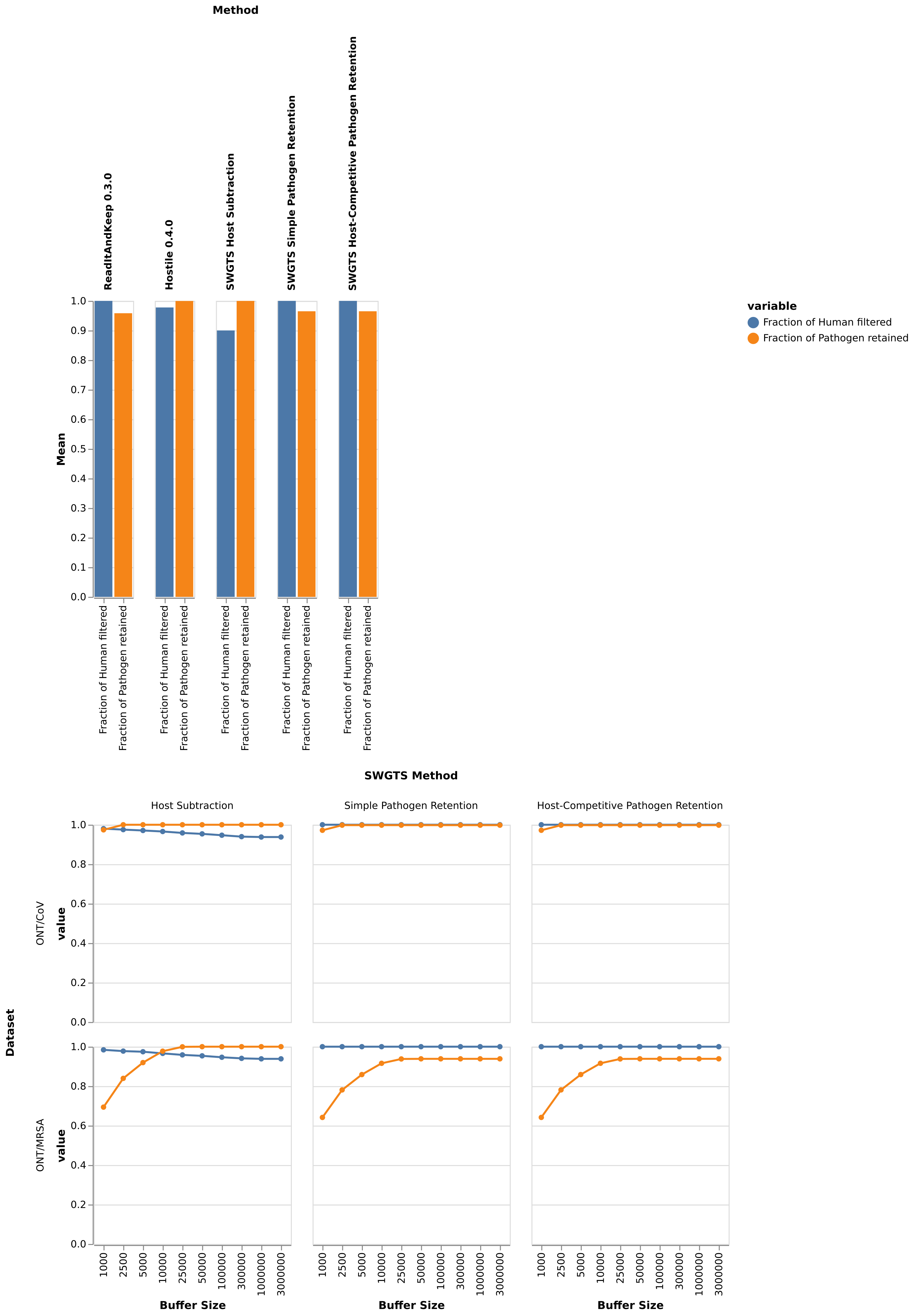

Supplement: btae332_Supplementary_Data [file btae332_supplementary_data.zip › Supplementary Figure 1. Filter Quality.png]
